# Woman and the Bicycle

**Published:** 1895-11

**Authors:** 


A Monthly Review of Medicine and Surgery.

*EDITORS:*

THOMAS LOTHROP, M. D. - - WM. WARREN POTTER, M. D.

All communications, whether of a literary or business nature, should be addressed to the managing editor: 284 Franklin Street, Buffalo, N. Y.

Vol. XXXV. NOVEMBER, 1895. No. 4.

### WOMAN AND THE BICYCLE.

THE subject given in the above title appears to be an absorbing one just now. The daily prints are full of interesting accounts showing the progress of this new fashion, not to say fad, and each in turn is enjoying, day by day, its own little joke, original or stale, at the expense of the sex. Medical societies, too, are discussing the question as to the propriety of the exercise, both from a moral and physical standpoint, and medical journals are commenting upon the action of the societies as well as discussing it editorially. We have refrained until now from entering the field, preferring to await the crystallisation of the crude and imperfect elements that are evolving from all this material.

The time, however, seems to have arrived when we may express an opinion on the merits of the question with a reasonable degree of intelligence. The usefulness of the bicycle for business purposes is beyond question. For the young woman, engaged in business pursuits, whose residence is remote from the place of her occupation, it affords unquestionably the best and healthiest method of transit. In its use she avoids the street cars in the early morning, at midday and at evening, when they are overcrowded, unhealthful, uncomfortable, even dangerous. In its use she reaches her business office refreshed by a ride in the early morning air, better prepared to cope with her duties, and it carries her home to luncheon or dinner with an appetite stimulated to sharpness and a digestion correspondingly improved. This is one of the practical views of this many-sided question.

The bicycle considered as a means of recreation takes us altogether into another field. Here we are obliged to take into account its moral and physical aspects. For a healthy woman whose social position is assured there is little to be said in criticism of her reasonable use of the wheel. Young girls, however, should be permitted to use it only under the strictest supervision of a competent parent or governess. The danger of its excessive use in youth is very great. No young girl should be permitted to ride to fatigue or to sit upon it otherwise than erect. Under no circumstances should she be permitted to ride at evening with the opposite sex unaccompanied by a proper chaperone. We have no doubt that a violation of this cardinal principle, that should be formulated as an inexorable rule, has been fruitful of much social mischief and would easily lead to ruinous consequences.

For the invalid, the question of the employment of the wheel becomes a problem of serious import. Physicians will constantly advise it on the broad ground of its furnishing healthful exercise in the open air, which, it must be confessed, is a strong argument, applicable to the needs of most semi-invalid women. Other physicians will condemn the use of the wheel *in toto*, on the narrow ground that no modest woman should permit herself to appear in public astride a bicycle; and especially should no woman out of health ride a wheel. The fact is, there are two sides to this question, as there are to most others, and the truth lies somewhere between these two extremes.

In prescribing the bicycle for an invalid, a physician should exercise the utmost caution and invoke his best judgment. Even then he must be prepared for disappointment. He must possess a perfect knowledge of a woman's physical condition; especially must he be familiar with the state of her pelvic and thoracic organs. It would be the idlest folly to direct a woman suffering from pus tubes to ride a bicycle in the expectation of benefit therefrom. It would be worse, even almost criminal, to recommend a woman with certain heart lesions to adopt the wheel as a cure. So, too, it may be said with reference to most pulmonic affections.

On the other hand, there are many disorders, or departures from the health line, that may be expected to derive undoubted benefit from a discreet use of the bicycle. Stout, plethoric, lithemic women, and all those whose sedentary habits tend to aggravate many neurotic affections, belong to this class. Some disorders of digestion, associated with chronic constipation, ought to derive benefit from this exercise. Since, however, it is our purpose to generalise, and not to particularise, we may only hint in the lines of indication and contra-indication of treatment.

Finally, we approach the most difficult part of the problem to deal with—namely, that of dress. There are many points relating to the fashion of the bicycle costume that every woman will regulate according to her own fancy or caprice. Each will decide for herself whether she will adopt bloomers, wear knickerbockers or the short loaded skirt. Neither of these points pertains to matters of health, provided only the fabric is adapted to the climate and the season of the year. The professional rider will naturally adopt some form of bloomer, or, perhaps, tight-fitting trousers, with short coat. The element of safety must enter into her choice of a costume. The amateur rider, however, if she be a woman of social position, will, naturally, choose the short skirt reaching to the boot tops and properly loaded to resist air currents and to protect her in case she is dismounted. Knickerbockers are becoming to young girls and possess some conveniences for the tourist. Whatever costume is chosen, stout leggings, reaching to the knee, should always be worn. That portion of a woman's bicycle apparel with which it is our province as physicians especially to deal is the body underwear. Every rider should wear woolen next the skin, regulating the weight according to season, and, preferably, the union undergarments should be chosen. Woolen absorbs moisture and prevents or limits the danger of taking cold. Lastly, the corset should be discarded and a corded flexible waist substituted. The latter furnishes a good support for the clothing and avoids undue pressure on the pelvic organs. Stout women, or those with pendulous abdomens, may, with great propriety, wear a well-adjusted abdominal band or supporter.

Every woman should carry a good woolen coat, which may be strapped to the handle-bar when not needed. She should also wear stout, well-fitting boots, and we think good cloth undertrousers would be a sensible addition to her costume.

And this is about the sum and substance of the whole case.

